# Autoantibodies in the pathogenesis of idiopathic inflammatory myopathies: Does the endoplasmic reticulum stress response have a role?

**DOI:** 10.3389/fimmu.2022.940122

**Published:** 2022-09-15

**Authors:** Esther Guadalupe Corona-Sanchez, Erika Aurora Martínez-García, Andrea Verónica Lujano-Benítez, Oscar Pizano-Martinez, Ivette Alejandra Guerra-Durán, Efrain Chavarria-Avila, Andrea Aguilar-Vazquez, Beatriz Teresita Martín-Márquez, Kevin Javier Arellano-Arteaga, Juan Armendariz-Borunda, Felipe Perez-Vazquez, Ignacio García-De la Torre, Arcelia Llamas-García, Brenda Lucía Palacios-Zárate, Guillermo Toriz-González, Monica Vazquez-Del Mercado

**Affiliations:** ^1^ Instituto de Investigación en Reumatología y del Sistema Músculo Esqueletico, Departamento de Biología Molecular, Centro Universitario de Ciencias de la Salud, Universidad de Guadalajara, Guadalajara, Mexico; ^2^ Departamento de Fisiología, Centro Universitario de Ciencias de la Salud, Universidad de Guadalajara, Guadalajara, Mexico; ^3^ Universidad de Guadalajara-Cuerpo Académico (UDG-CA)-703, Inmunología y Reumatología, Centro Universitario de Ciencias de la Salud, Universidad de Guadalajara, Guadalajara, Mexico; ^4^ Doctorado en Ciencias Biomedicas, Centro Universitario de Ciencias de la Salud, Universidad de Guadalajara, Guadalajara, Mexico; ^5^ Departamento de Morfología, Centro Universitario de Ciencias de la Salud, Universidad de Guadalajara, Guadalajara, Mexico; ^6^ Departamento de Disciplinas Filosófico Metodológicas e Instrumentales, Centro Universitario de Ciencias de la Salud, Universidad de Guadalajara, Guadalajara, Mexico; ^7^ Hospital Civil de Guadalajara “Dr. Juan I. Menchaca”, Especialidad de Medicina Interna, Padrón Nacional de Posgrados de Calidad (PNPC) Consejo Nacional de Ciencia y Tecnología (CONACyT), Guadalajara, Mexico; ^8^ Instituto de Biología Molecular en Medicina, Universidad de Guadalajara, Centro Universitario de Ciencias de la Salud, Guadalajara, Mexico; ^9^ Escuela de Medicina y Ciencias de la Salud, Tecnológico de Monterrey, Zapopan, Mexico; ^10^ Departamento de Inmunología y Reumatología, Hospital General de Occidente y Universidad de Guadalajara, Guadalajara, Mexico; ^11^ Hospital Civil de Guadalajara “Dr. Juan I. Menchaca, ” Especialidad de Reumatología, Padrón Nacional de Posgrados de Calidad (PNPC) Consejo Nacional de Ciencia y Tecnología (CONACyT), Guadalajara, Mexico; ^12^ Instituto Transdisciplinar de Investigación y Servicios (ITRANS), Universidad de Guadalajara, Zapopan, Mexico

**Keywords:** endoplasmic reticulum stress, idiopathic inflammatory myopathies, myositis specific antibodies, autophagy, myositis associated antibodies

## Abstract

Idiopathic inflammatory myopathies (IIMs) are a group of rare, acquired autoimmune diseases characterized by profound muscle weakness and immune cell invasion into non-necrotic muscle. They are related to the presence of antibodies known as myositis-specific antibodies and myositis-associated antibodies, which are associated with various IIM phenotypes and the clinical prognosis. The possibility of the participation of other pathological mechanisms involved in the inflammatory response in IIM has been proposed. Such mechanisms include the overexpression of major histocompatibility complex class I in myofibers, which correlates with the activation of stress responses of the endoplasmic reticulum (ER). Taking into account the importance of the ER for the maintenance of homeostasis of the musculoskeletal system in the regulation of proteins, there is probably a relationship between immunological and non-immunological processes and autoimmunity, and an example of this might be IIM. We propose that ER stress and its relief mechanisms could be related to inflammatory mechanisms triggering a humoral response in IIM, suggesting that ER stress might be related to the triggering of IIMs and their auto-antibodies’ production.

## 1 Introduction

**Figure 1:**
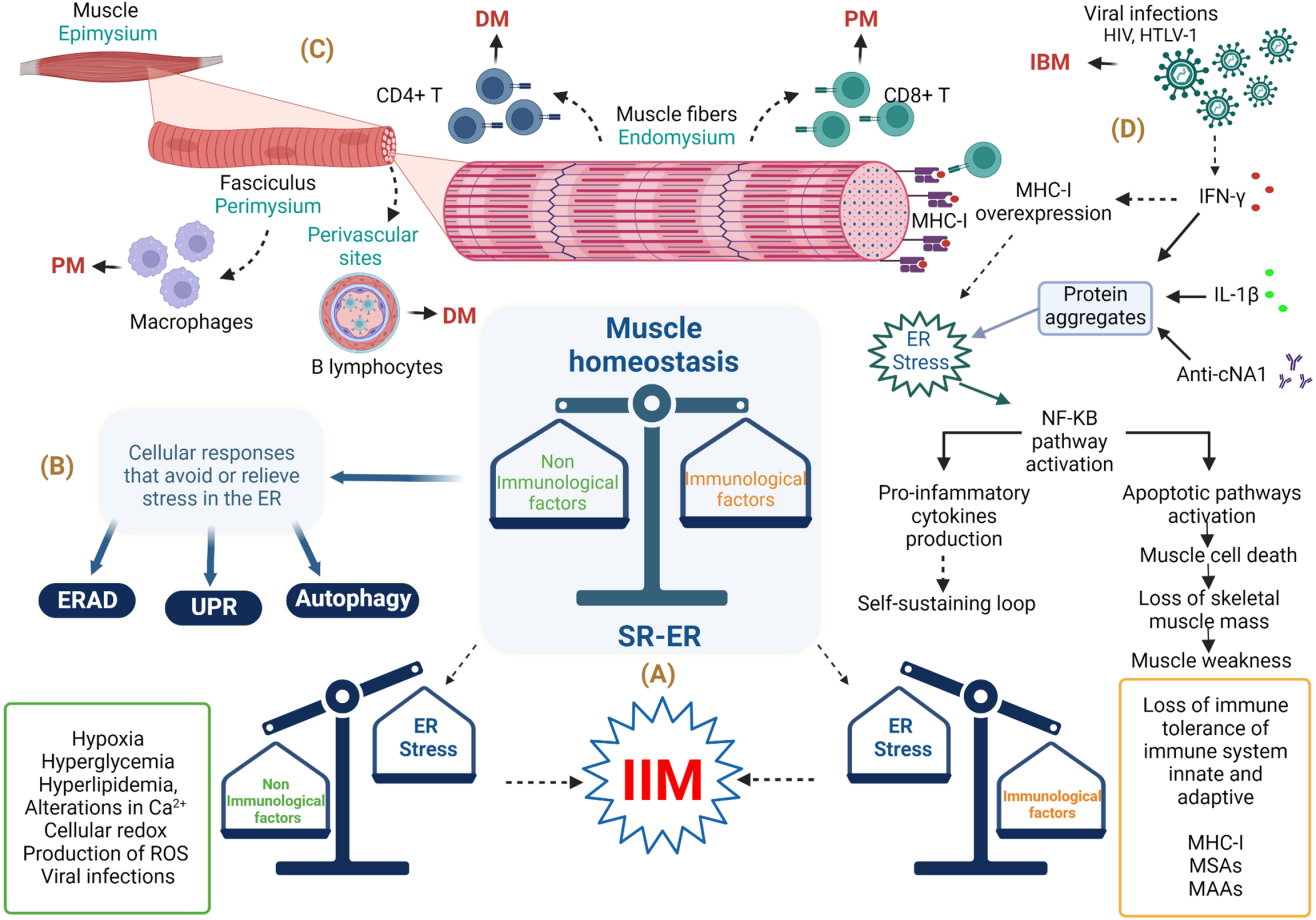
Involvement of endoplasmic reticulum stress in the musculoskeletal system in idiopathic inflammatory myopathies. **(A)** Immunopathological and non-immunopathological factors with possible repercussions on homeostasis of the musculoskeletal system. **(B)** ER stress relief mechanisms. **(C)** Histological importance in the diagnosis of IIM. **(D)** Involvement of immune and non-immune responses in IBM*. Abbreviations:* anti-cN1A, anti-cytosolic 5’-nucleotidase 1A antibodies; DM, dermatomyositis; ER, endoplasmic reticulum; ERAD, endoplasmic reticulum–associated protein degradation; HIV, human immunodeficiency virus; HTLV-1, human T lymphotropic virus; IBM, inclusion body myositis; IFN-γ, interferon gamma; IIM, idiopathic inflammatory myopathy; IL-1β, interleukin-1 beta; MHC-1, major histocompatibility complex class I; MSAs, myositis-specific antibodies; MAAs, myositis-associated auto-antibodies; NF-KB, nuclear factor κB; PM, polymyositis; ROS, reactive oxygen species; SR-ER, sarcoplasmic reticulum–endoplasmic reticulum; UPR, unfolded protein response. The figure was created with BioRender.com (agreement no. ZO24BHA4GB).

Idiopathic inflammatory myopathies (IIMs), also known as myositis, are a group of conditions characterized by chronic inflammation of the musculoskeletal system that leads to proximal or distal muscle weakness, although other organs such as the skin, joints, heart, lungs, and gastrointestinal tract can also be affected (1). The IIM pathogenesis includes genetic, environmental, and immune factors (2, 3). To date, the immunopathological mechanisms of this group of conditions remain incompletely understood; however, they are related to inflammatory responses characterized by the infiltration of T- and B-cells in muscle tissue, the presence of myositis-specific antibodies (MSAs) myositis-associated auto-antibodies (MAAs), and ubiquitous abnormal overexpression of major histocompatibility complex class I (MHC-I) in myofibers (2) (**Figure 1A**). However, one of the clinical observations is that the level of muscle inflammation does not correspond to the severity of the disease or the alterations in muscle fibers in patients with IIM, so non-immunological mechanisms are involved (4). Among the non-immunological mechanisms involved in the pathogenesis of IIM are endoplasmic reticulum (ER) stress and the responses that avoid or relieve this stress, such as the unfolded protein response (UPR), ER-associated protein degradation (ERAD), and autophagy (**Figure 1B**). Specifically, the UPR increases the capacity of the ER to fold proteins efficiently and attenuates the general translation of proteins to reduce the load on the ER, while proteins that cannot be repaired are removed by ERAD and autophagy (5). It is important to emphasize that the ER is very sensitive to challenges that can compromise its structure, integrity, and function; such challenges include calcium (Ca^2+^) depletion, protein glycosylation, disulfide-bond formation, hypoxia, redox conditions, and viral infection, which can result in unfolded or misfolded protein accumulation, generating ER stress that triggers an inflammatory response (6–8) (**Figure 1A**). Currently, it is known that ER stress is involved in the pathogenesis of different diseases, such as obesity, diabetes, atherosclerosis, inflammatory bowel disease, Alzheimer’s disease, breast cancer, rheumatoid arthritis, Sjögren’s syndrome, and myopathies, among others (9, 10). This review will focus on current state-of-the-art research seeking to understand ER stress, focusing on UPR, ERAD, and autophagy as trigger factors in the IIM clinical phenotype pathogenesis and the possible link with MSAs.

## 2 Idiopathic inflammatory myopathies: Classification, diagnosis, and treatment

In 2017, the European League Against Rheumatism and the American College of Rheumatology (EULAR/ACR) published the most recent criteria for myositis classification for adult and juvenile IIM, which covered the following conditions: dermatomyositis (DM), amyopathic DM (ADM), juvenile DM (JDM), polymyositis (PM), immune-mediated necrotizing myopathy (IMNM), juvenile myositis (JM), and inclusion body myositis (IBM) (11). The clinical characteristics of IIM are proximal and distal muscle weakness, fatigue, fever, cutaneous features including pathognomonic rashes in DM (heliotrope, Gottron’s sign), dysphagia, increased serum muscle enzyme levels (of, e.g., aspartate aminotransferase, alanine aminotransferase, lactate dehydrogenase, and aldolase), muscle biopsy with histopathological features related to inflammation in the perimysium or perivascular areas, necrotic fibers between other characteristics, detection of conduction abnormalities by electromyography, and the presence of MSAs and MAAs (12). MSAs are used as a diagnostic tool in IIM; such auto-antibodies include anti-aminoacyl tRNA synthetase antibodies (anti-ARS), anti-nucleosome remodeling deacetylase antibodies (anti–Mi-2), anti-melanoma differentiation–associated protein 5 (anti-MDA5/CADM140), anti-nuclear matrix protein (anti-MJ/NXP2), anti-transcription intermediary factor-1 γ/α (anti-TIF-1 γ/α or p155/140, anti-hydroxymethylglutaryl coenzyme A reductase (anti-HMGCR), anti-small ubiquitin-like modifier activating (anti-SAE), anti-cytosolic 5’-nucleotidase 1A (anti-cN1A), and anti-signal recognition particle (anti-SRP) (13, 14) (**Table 1**). However, the anti-histidyl tRNA synthetase antibody (anti–Jo-1), an anti-ARS auto-antibody, is considered only by the EULAR/ACR 2017 guideline as a criterion for IIM classification (15). IIM treatment aims to reduce muscle inflammation and improve extra muscular manifestations, allowing patients to have a better quality of life, and it consists of high doses of oral glucocorticoids as initial treatment in combination with other conventional disease-modifying anti-rheumatic drugs or biological therapy (1).

**Table 1:** Stratification of Idiopathic Inflammatory Myopathies patients according to myositis-specific antibodies.

MSAs	Target	IIM
Anti-ARS	Aminoacyl tRNA synthetase	ASS
Anti-Jo-1	Histidyl tRNA synthetase	ASS
Anti-EJ	Glycyl tRNA synthetase	ASS
Anti-PL-7	Threonyl tRNA synthetase	ASS
Anti-OJ	Isoleucyl tRNA synthetase	ASS
Anti-PL-12	Alanyl tRNA synthetase	ASS
Anti-YRS/HA	Tyrosyl tRNA synthetase	ASS
Anti-KS	Asparaginyl tRNA synthetase	ASS
Anti-Zo	Phenylalanyl tRNA synthetase	ASS
Anti-cN1A	Cytosolic 5’-nucleotidase 1A	IBM
Anti-HMGCR	3-hydroxy-3-methylglutaryl coenzyme A reductase	IMNM
Anti-MDA5 (anti-CADM140)	Melanoma differentiation-associated protein 5	DM, ADM
Anti–Mi-2	Nucleosome remodeling deacetylase (Mi-2α/β)	DM
Anti–NXP-2 (anti-MJ)	Nuclear matrix protein 2	DM
Anti-SAE	Small ubiquitin-like modifier activating enzyme (SAE1/2)	DM
Anti-SRP	Signal recognition particle	PM, IMNM
Anti-TIF1γ/α (anti-p155/p140)	Transcription intermediary factor-1 γ/α	DM

ADM, amyopathic dermatomyositis; anti-ARS, anti-aminoacyl tRNA synthetase antibodies; anti–Jo-1, anti-histidyl tRNA synthetase antibodies; anti-EJ, anti-glycyl tRNA synthetase antibodies; anti–PL-7, anti-threonyl tRNA synthetase antibodies; anti-OJ, anti-isoleucyl tRNA synthetase antibodies; anti–PL-12, anti-alanyl tRNA synthetase antibodies; anti-YRS/HA, anti-tyrosyl tRNA synthetase antibodies; anti-KS, anti-asparaginyl tRNA synthetase antibodies; anti-Zo, anti-phenylalanyl tRNA synthetase antibodies; anti-cN1A, anti-cytosolic 5’-nucleotidase 1A antibodies; anti-HMGCR, anti-hydroxymethylglutaryl coenzyme A reductase antibodies; anti-MDA5/CADM140, anti-melanoma differentiation–associated protein 5 antibodies; anti–Mi-2, anti-nucleosome remodeling deacetylase Mi-2 α/β antibodies; anti-MJ/NXP2, anti-nuclear matrix protein 2 antibodies; anti-SAE, anti-small ubiquitin-like modifier activating antibodies; anti-SRP, anti-signal recognition particle antibodies; anti-TIF-1γ/α or p155/140, anti-transcription intermediary factor 1 α/γ antibodies; ASS, anti-synthetase syndrome; DM, dermatomyositis; IBM, inclusion body myositis; IIM, Idiopathic Inflammatory Myopathies; IMNM, immune-mediated necrotizing myopathy; JDM, juvenile dermatomyositis; JM, juvenile myositis; MSAs, myositis-specific antibodies; PM, polymyositis.

## 3 Clinical features in idiopathic inflammatory myopathies

### 3.1 Dermatomyositis

This phenotype is characterized by the presence of heliotrope, a pathognomonic cutaneous manifestation that involves the presence of a bilateral and symmetrical edema with violaceous coloration in the upper eyelids (16) as well as the presence of other cutaneous rashes, including Gottron’s sign, V sign, and Shawl’s sign (17). Histopathology shows predominant CD4^+^/T-cell perivascular infiltration (18). In classical adult DM, the enzyme muscle level rises significantly, but it usually carries a good clinical prognosis. This phenotype of IIM has been associated with the presence of anti–Mi-2 and anti-MJ/NXP2 (1, 13).

### 3.2 Amyopathic dermatomyositis

These patients present the same cutaneous manifestations observed in classic DM; however, there is no clinical or laboratory evidence of muscle disease (19). This phenotype of IIM has been broadly associated with anti-MDA5/CADM140 auto-antibodies and the development of rapidly progressive interstitial lung disease (13).

### 3.3 Juvenile dermatomyositis

This DM variant includes the same features as those of classic DM; however, it develops during childhood or youth. This phenotype has a greater association with calcinosis, pericarditis, and gastrointestinal ulcers (20, 21).

### 3.4 Polymyositis

PM is observed as a kind of proximal muscle weakness without dermatologic manifestation found usually in the adult population. Histopathology shows predominant CD8^+^/T-cell perivascular infiltration and upregulation of MHC-I molecules in muscle biopsy (18). PM patients are usually seronegative for MSAs or MAAs, and they are now considered a rare IIM subgroup; in a study of a U.K. IIM cohort of 255 patients, PM was diagnosed in 37/255 (14.5%) patients, but reclassification using the EULAR/ACR 2017 criteria led to the final diagnosis of only 9/255 (3.5%) patients, with the rest meeting criteria for IMNM, DM, overlap syndromes, etc. (22). This phenotype of IIM might be associated with anti-synthetase syndrome, and patients are usually positive for anti-ARS auto-antibodies, especially anti–Jo-1 (13, 23).

### 3.5 Immune-mediated necrotizing myopathy

The necrotizing terminology in this context is used to describe muscle fiber necrosis with minimal leukocyte infiltrates, which could be due to genetic causes or statin-induced (24). The muscular weakness found in these patients progresses more rapidly and tends to be more severe. Another important features are the serum levels of creatine phosphokinase, which are remarkably high in this phenotype of IIM compared to other types of myositis, and the presence of anti-SRP or anti-HMGCR auto-antibodies (25, 26).

### 3.6 Juvenile myositis

This myositis phenotype is diagnosed according to the EULAR/ACR 2017 guideline when the first symptom occurs at <18 years of age and there are no cutaneous manifestations (the major differential feature of JDM) (15).

### 3.7 Inclusion body myositis

IBM patients experience a slow progression of skeletal–muscle disease, usually after 50 years of age, with a higher prevalence in men and a predominance of distal muscle weakness. In addition, dysphagia occurs in >50% of IBM patients (1). Serum detection of anti-cN1A is highly specific for IBM (90%–95%) compared to DM or PM (5%–10%); however; this auto-antibody has also been detected in patients with systemic lupus erythematosus (0%–20%) and Sjögren’s syndrome (0%–36%) (27).

## 4 Pathological mechanisms in idiopathic inflammatory myopathies

The common denominator in all IIMs is muscle inflammation (28). Muscle fibers have a cylindrical structure and consist of intercalating thick and thin filaments (myofilaments) that are organized longitudinally in sarcomeres that allow the contraction of muscle fibers (29). In particular, muscle fibers are arranged in three layers of connective tissue, as follows (a) the endomysium, which surrounds the muscle fibers; (b) the perimysium; and (c) the epimysium, which is composed of fasciculi surrounding the entire muscle (30). There are no conclusive data on the pathophysiological mechanisms related to muscle inflammation in IIM, where genetic factors such as human leukocyte antigen classes I and II (HLA-I and HLA-II) and environmental factors such as ultraviolet radiation and viral infections play a role (31–33). However, these do not fully explain the triggering of IIM, so the participation of immunopathological mechanisms related to the innate and adaptive immune systems are probably linked to IIM development (1). Nevertheless, as we have mentioned in previous paragraphs, the level of muscle inflammation does not correspond to the severity of the disease or to alterations in muscle biopsy in IIM; therefore, these pre-suppose the contribution of non-immunological mechanisms such as ER stress and altered proteins responses (UPR, ERAD, and autophagy). Based on this information, it is impossible to rule out the idea that the immunological mechanisms do not have repercussions on the non-immunological ones.

## 5 Immunopathological mechanisms in idiopathic inflammatory myopathies

The involvement of both the innate and the adaptive immune systems has been reflected in histopathological evidence characterized by the infiltration of inflammatory cells in the muscle and skin, microvasculature affection, auto-antibody production against nuclear and cytoplasmic proteins, and inflammatory responses by interferon signature (34). For example, in DM patients, a complement-mediated micro-angiopathy that affects blood vessels of muscle tissue has been described; resulting in complement activation and membrane attack complex formation that leads to perivascular inflammation, capillary necrosis, and ischemic damage of myofibers; along with pro-inflammatory cytokines, macrophages, a high percentage of CD4^+^ T and B lymphocytes in perivascular sites, and dendritic plasmacytoid cells (18, 35, 36). The immune response in PM is mediated by lymphocytic infiltrates with a predominance of CD8^+^ T lymphocytes related to perforin and granzyme release in the endomysium as well as a lower proportion of macrophages in perivascular sites; increased expression of MHC-I; the roles of interleukin (IL)-1 and interferon-γ (IFN-γ) in myotoxicity; chronic inflammation and fibrosis *via* the involvement of transforming growth factor-β (TGF-β); and T-cell extravasation in muscle tissue through the involvement of chemokines like C-X-C motif chemokine ligand 8 (CXCL8), C-X-C motif chemokine ligand 9 (CXCL9), C-C motif chemokine ligand 2 (CCL2), and C-C motif chemokine ligand 9 (CCL9) (18, 36, 37) (**Figure 1C**). It has been observed that muscle cells also express human leukocyte antigen-G (HLA-G) in IIM due to stimulation by IFN-γ, which is synthesized locally by inflammatory cells of the disease-specific immune micro-environment, and the increase of HLA-G can interfere with cytotoxic effector functions of CD8^+^ T- and natural killer (NK) cells (38) because this molecule is a ligand for killer cell Ig-like receptor 2DL4 (KIR2DL4, also known as CD158d), an inhibitor receptor expressed in these cells (39). The immunopathological mechanisms involved in IMNM are mainly associated with statin prescription and the presence of anti-SRP and/or anti-HMGCR auto-antibodies; further ectopic expression of the respective auto-antigens in the myofiber surface has been reported (35, 40, 41). In addition, these auto-antibodies can induce muscle atrophy incrementally in the transcription of genes related to atrophy—such as muscle atrophy F-box (*MAFbx*) and tripartite motif containing 63 (*TRIM63*)—and reactive oxygen species (ROS) and can decrease the production of anti-inflammatory cytokines such as IL-4 and IL-13 (26, 42). Particularly, IBM is considered a complex disorder involving inflammatory and cytotoxic responses mediated by CD8^+^ T-cells with vacuole formation, accumulation of tubulofilamentous inclusions, and cytoplasmic accumulations of amyloid filaments, which could trigger or exacerbate ER stress responses (43). The IBM pathogenesis includes mitochondrial dysfunction as reflected by high levels of differential growth factor 15 (GDF15), a mitochondrial disease marker (44). Furthermore, in myoblasts of IBM patients, reduced adenosine triphosphate production, cellular vulnerability to oxidative stress, and reduced mitochondrial size have been documented (44). The mitochondrial damage in IBM is also associated with impaired autophagy mechanisms, and abnormal autophagy causes autophagosome accumulation with vacuole formation (27).

## 6 Non-immunopathological mechanisms

The cell has an integrated and interconnected signaling system that avoids or relieves ER stress through processes such as UPR, ERAD, and autophagy, which are considered to be non-immunological mechanisms associated with IIM pathogenesis (45, 46). The fundamental goal of these mechanisms is to recover ER homeostasis to preserve ER functions that are important for cell survival (47). Briefly, we describe some important mechanisms to achieve this goal.

### 6.1 Endoplasmic reticulum

An organelle associated with skeletal muscle homeostasis is the sarcoplasmic reticulum (SR), which is part of compartmentalization in the cytoplasm of the eukaryotic cell by an endomembrane system (ES) (48–50). The functioning of the ES can be represented as a factory, where proteins assembly begins; components are then delivered to the Golgi apparatus (GA), where the protein assembly ends, and the proteins are modified, labeled, classified, and finally packaged to be sent to their destination. The cell nucleus coordinates this entire manufacturing process in communication with ES through transport vesicles that bud from the membrane (donor) to merge with the membrane of the next compartment (acceptor), giving rise to what is known as the secretory pathway (51–53). Because there are multiple actors in the production of proteins, coordination between them is crucial to ensure protein synthesis occurs with an adequate structure and function, and one of these main actors is the ER (54, 55). The ER is involved in many cellular functions such as synthesis and processing (folding, maturation, and post-translational modification) of proteins (56–58). The ER is divided into three domains that are functionally and structurally different (56). The first domain includes a nuclear envelope, which forms using inner and outer membranes, which are continuous to the nuclear pores and function as a site where the proteins of the membrane are diffused through the nucleus and cytoplasm (56, 59). The second domain represents a smooth and rough ER (with attached ribosomes) (56). The third domain involves numerous heterotypic membrane contact sites with membranes of other organelles, such as the plasma membrane ER–plasmatic membrane, which is a classic example of an heterotypic membrane contact site and the first such example described in muscle cells related to muscle contraction through a massive influx of Ca^2+^ (60), ER–mitochondria, ER–peroxisomes, ER–lipid droplets, and ER–Golgi (61, 62). The secretory pathway can be explained in the following steps: (a) synthesis, anchoring, and translocation of the protein to ER lumen; (b) protein folding and quality control; and (c) protein sorting (63).

#### 6.1.1 Synthesis, anchoring, and translocation of the protein to the endoplasmic reticulum lumen

**Figure 2:**
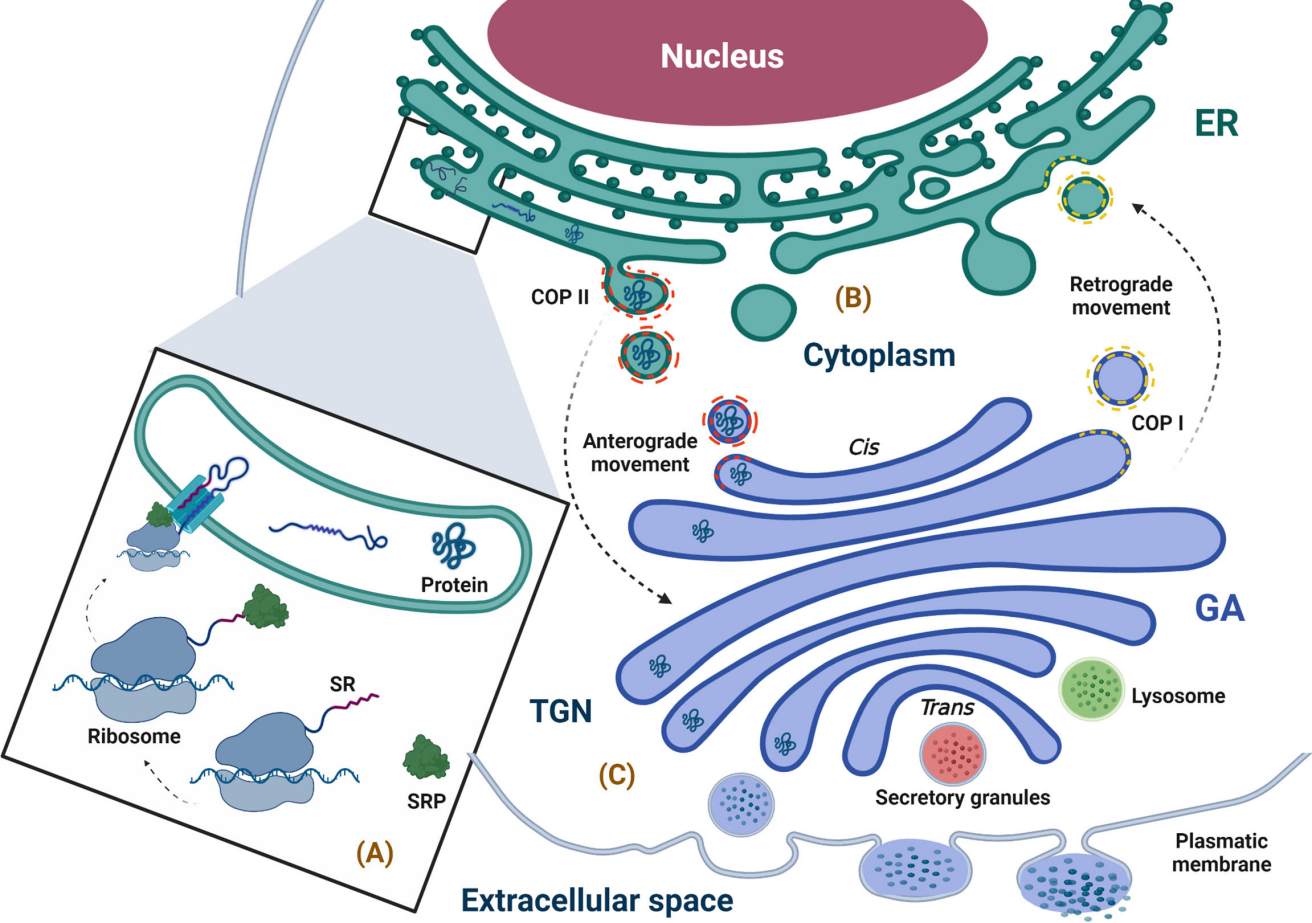
Physiological overview of the secretory pathway. **(A)** Synthesis, anchoring, and translocation of protein to the ER (ribosome binding to the ER membrane is promoted by the SRP complex, which recognizes the SR; once in the ER lumen, the ribosome–nascent protein complex, coupled to the translocon, translates the polypeptide into the lumen of the ER), **(B)** Protein folding and quality control (the newly synthesized proteins with correct folding and assembly continue their forward movement in COPII-coated vesicles that fuse with the GA for cargo delivery; however, the secretory pathway is bidirectional, and the circulatory pathway may stretch from the GA to ER, moving retrogradely through COPI-coated vesicles). **(C)** Protein sorting (when proteins arrive at the TGN, they are classified and packaged in transport vesicles to be sent to their final destination, i.e., the extracellular space). ER, endoplasmic reticulum; GA, Golgi apparatus; SR, signal recognition; SRP, signal recognition particle; TGN, trans-Golgi network. The figure was created with BioRender.com (agreement no. AM24BH3QGW).

When the proteins are synthesized by ribosomes attached to the ER membrane, they are translocated to the ER while their translation is in progress (co-translational manner), and these proteins will either follow the conventional secretory pathway or exist inside the ER. If free ribosomes synthesize proteins in the cytoplasm, they can be directed to different organelles, such as the nucleus, mitochondria, or peroxisomes, and can also enter the ER when their translation is complete in a process called post-translational translocation (64). The ribosomes attached to the ER membrane are responsible for synthesis in approximately 1/3 of total cellular proteins, and it is well known that this attachment is promoted by the SRP complex, which consists of 7SL RNA and six different polypeptides that are named according to their molecular mass in kilodaltons (kDa), as follows: SRP9, SRP14, SRP19, SRP54, SRP68, and SRP72 (65–68). The SRP complex recognizes a sequence of hydrophobic amino acids in the N-terminal region of the nascent protein known as a signal sequence or leader sequence through SRP54 kDa (65, 69–74). When protein translation begins in the cytoplasm, the signal sequence is exposed, and the SRP complex recognizes it through SRP19 kDa, preventing the continuation of the translation (arrest elongation) (65, 67, 75–77). Finally, the SRP complex is attached to its receptor through a heterodimer formed by SRP68 and SRP72 kDa, allowing the nascent protein–ribosome complex to be coupled to the translocation site, which is a protein channel known as translocon (74, 76, 78–80). This event permits the continuation of protein translation with the subsequent translocation of the polypeptide to the ER lumen through translocon while the ribosome is still synthesizing it or with insertion in the ER membrane (65, 67, 81–84) (**Figure 2A**).

#### 6.1.2 Protein folding and quality control

Once in the ER, the folded protein is modified and exposed to quality control in order to achieve its native structure (proper three-dimensional conformation) through co-translational modifications such as N-glycosylation and disulfide bond formation (68, 82, 85, 86). Importantly, those proteins that undergo quality control in the ER to confirm their correct folding and assembly are packaged in a coat complex protein (COP)II–coated vesicle, which allows its transport through the secretory pathway by an anterograde movement (forward transport) to the next compartment of this pathway, i.e., the GA (48, 87). Furthermore, traffic through the secretory pathway is bidirectional; transportation from the GA to the ER is a retrograde movement allowing immature or ER-resident proteins to go back to ER through COPI-coated vesicles produced in the trans-Golgi network (TGN) and involved in traffic between GA compartments (88) (**Figure 2B**). In this way, the ER is not only responsible for the synthesis of proteins that follow the secretory pathway but also for their processing and maturation and is essential for shipment to its destiny (89–91).

Multiple factors assist in the folding and maturation process of newly synthesized proteins, such as ER chaperone proteins and folding enzymes (92, 93). The best-recognized ER chaperones are the glucose-regulated/immunoglobulin-binding protein of 78 kDa (BiP/GRP78), calnexin (CNX), and calreticulin (CRT) (93–101).

#### 6.1.3 Protein sorting

Within the GA, proteins continue their maturation; the N-linked glycan chains (preformed oligosaccharides) added to the peptides in the ER are structurally modified through a series of reactions that occur in a sequential manner *via* multiple trimming and elongation steps (102). The labeled proteins with their specific N-linked glycan chains, when they arrive to the TGN, are classified and packaged in transport vesicles to be sent to their final destination in the cellular surface, extracellular medium, or any of the compartments of the secretory pathway (53, 101, 103) (**Figure 2C**).

### 6.2 Endoplasmic reticulum stress

The ER is very sensitive to cellular disturbances that can disrupt the efficiency of its function, including a loss of Ca^2+^ homeostasis, impaired redox balance and endogenous ROS production, nutrient deprivation, virus infection, changes in protein glycosylation and autophagy defects, protein folding defects, or an increase in protein synthesis level (104). These cellular disturbances can influence protein synthesis and folding, including post-translational modifications, and lead to an accumulation of unfolded and/or misfolded proteins in the ER lumen, generating a cellular stress situation known as ER stress (6, 8, 57, 105).

### 6.3 Unfolded protein response

The best-known response to relieve ER stress caused by the detection of unfolded and/or misfolded proteins in the ER lumen is the UPR pathway (106) (**Figure 1B**). UPR transduces the stress signal from the ER to the cell nucleus through three sensors, which are the transmembrane proteins inositol-requiring kinase 1 (IRE1), double-stranded RNA–activated protein kinase (PKR)-like ER kinase (PERK), and activating transcription factor 6 (ATF6) (68, 107, 108). The N-terminal luminal domain of these three sensors is responsible for detecting the accumulation of unfolded/misfolded proteins in the ER lumen (109). BiP/GRP78 is a repressor of UPR when it forms a stable complex with the luminal domain of the three sensors in unstressed cells; however, high concentrations of unfolded/misfolded proteins compete for BiP/GRP78, blocking its attachment to the three sensors (competition model) (110–114).

IRE1 is a type I transmembrane protein resident in the ER; PERK is also an ER resident type I transmembrane protein with serine/threonine kinase activity (115). Under conditions of ER stress, PERK can activate nuclear factor (NF)-κB in a manner dependent on the control of the translation mediated by the phosphorylation of eIF2α and independent of I kappa B kinase (IKK) activation, respectively (116). Finally, ATF6 is a type II transmembrane protein whose C-terminal domain is pointed toward the lumen of the ER and the N-terminal domain to the cytosol (117–119). Following stress-induced dissociation of BiP/GRP78 from ATF6 in the ER, it travels through COPII transport vesicles to the GA, where it undergoes proteolytic processing (120, 121).

### 6.4 Endoplasmic reticulum-associated protein degradation

ERAD is a crucial mechanism for maintaining protein quality control in the ER. It is responsible for removing misfolded proteins and redirecting them to the cytosol to be removed by the proteasome (122–124). Given the above, it is clear that the proteasome cannot process protein aggregates, so the cell has another important intracellular proteolytic system called autophagy (125).

### 6.5 Autophagy

Autophagy is an important catabolic process of cell degradation and macromolecule recycling (mainly damaged/old organelles and protein aggregates) (126). It is constitutively carried out at basal levels to maintain cellular homeostasis, but the level of autophagy can markedly increase under stress conditions as a cytoprotective response (127). Three types of autophagy are known to exist, including micro-autophagy, macro-autophagy, and chaperone-mediated autophagy, and all culminate in lysosome-mediated cargo degradation but differ in the mechanisms through which they deliver cargo to lysosomes (128). Macro-autophagy is the most studied process for the degradation of large loads, such as damaged organelles and protein aggregates; when speaking of autophagy, macro-autophagy is typically what is being referred to (129). The cellular hallmark of autophagy is the formation of double-membrane structures called autophagosomes that are responsible for transporting cargo to lysosomes (130).

To deliver cargo to the lysosome, the autophagosome fuses with the lysosome through its outer membrane, leading to the formation of an autolysosome; lysosomal enzymes first break down the inner membrane of the autophagosome to release the autophagic cargo and then break it down into its components, which are returned to the cytoplasm *via* the lysosomal membrane, allowing for their re-use by the cell (131). It has been reported that autophagosomes are composed of membranes from multiple sources, so the ER could be one of several sources (132).

Autophagy is mediated by a group of approximately 30 evolutionary conserved autophagy-related genes (ATGs), many of which have been detected in mammalian homologs. Among them, ATG1–10, 12–14, 16–18, 29, and 31 are essential for autophagosome formation (133). Autophagy can be selective or non-selective. Selective autophagy consists of labeling the cargo to be sequestered using ubiquitin (134). Subsequently, autophagy receptors such as ubiquitin-binding protein p62, also called sequestosome 1 (SQSTM1), recognize the marked charge and function as adapters that, by associating with certain specific proteins of the inner membrane (proteins of the ATG8 family) of the phagophore (early stage of autophagosome formation), can connect the cargo labeled with the inner membrane of the growing phagophore to allow for phagophore formation (135).

### 6.6 Endoplasmic reticulum/endoplasmic reticulum stress in idiopathic inflammatory myopathies

The IIM pathogenesis is associated with immunological and non-immunological mechanisms, which involve the participation of autoreactive T lymphocytes and auto-antibody production. Despite these, the literature has not explained the origin of muscle fiber damage or muscle weakness (136). To date, the most well-described example is upregulation of the expression of MHC-I in myofibers (sarcolemma surface), whose expression is not usual in normal muscle tissue (endomysial capillaries), thus stimulating the activation of ER stress responses such as an accumulation of misfolded glycoproteins and the activation of NF-κB, causing an inflammatory response *via* IRE1, tumor necrosis factor (TNF)-α, and TRAF2 (137, 138). It has been demonstrated that BiP/GRP78, CRT, and heat shock protein 90-α2 expression levels are augmented according to intermediate or high MHC-I expression in myositis muscle (139). In addition, elevated expression of several genes, including *PERK* and *ATF3*, in myositis, has been observed. Furthermore, the PERK signaling *via* transcription factor CCAAT/enhancer binding protein δ (C/EBPδ) has been associated with tumors by the action of chemokines such as CXCL8, and C-C motif chemokine ligand 20 (CCL20), which have been related to tumor-promotion with immunosuppressive properties and are triggered by activated oncogenes, nutrient deprivation, and hypoxia (140). In addition, the increased expression of PERK has been correlated with macrophage and dendritic cell infiltration into the tumor micro-environment (141). At this time, the link between IIM and cancer development is not well understood (142), although factors strongly associated with cancer development are anti-TIF-1γ and anti-MJ/NXP2 (143). In addition, our research group reported the presence of MSAs, particularly anti–TIF-1γ, in a group of women with breast cancer without clinical evidence of myositis (144). We will next address the three points of this section.

As mentioned above, the ER stress response pathway activates NF-κB. Interestingly, it has been shown that caspase-12, the main caspase associated with the apoptosis pathway, is highly expressed in mouse skeletal muscle tissues, so the activated apoptotic pathways in muscle cells could induce cell death, a loss of skeletal muscle mass, and muscle dysfunction and weakness (4).

It has also been reported that TRE-H-2Kb x mck-Tta (HT) mice with conditional overexpression of H-2Kb in muscle presented an upregulation of ER stress markers and molecular targets of UPR such as BiP/GRP78, CRT, CNX, and ATF6, in addition to pathologic features of non-specific myopathy with variations in myofiber size, numerous centronucleated fibers, and endomysial fibrosis (139). In that work, the authors also reported that an intracellular accumulation of MHC-I in the muscle of IBM patients correlated with UPR (139). Such findings suggest that MHC-I involvement in myositis is pathological because of the triggering of UPR responses instead of the facilitation of antigen presentation to CD8^+^ T lymphocytes. Therefore, the detection of MHC-I by immunostaining may be a diagnostic approach in IIM (145). However, although the positive detection of MHC-I has a high sensitivity for IIM, it has a low specificity, hence, it has been suggested that it can be combined with the positive detection of MHC-II (146).

In a study of our work group, we reported that recombinant human IL-1β induced phosphorylation and upregulation of SRP72 in Jurkat cells (147). Importantly, this cytokine and IL-18 are overexpressed in muscle biopsies of patients with DM and PM (148, 149). IL-1β and IL-18 are proteolytically matured by the NLRP3 inflammasome and allow the recruitment and maturation of caspase-1 (150). Another mechanism linked to the NLRP3 inflammasome is pyroptosis, a kind of cellular death, in addition to apoptosis and NETosis, which are also sources of auto-antigens related to the inflammatory response in the immunopathogenesis of autoimmune diseases such as IIM (151). In IMNM, an isolated report showed a correlation of BiP/GRP78 in muscle biopsy assessed by immunohistochemistry and serum lactate dehydrogenase along with a negative correlation with the Manual Muscle Testing–8 score (152).

Aggregates of p62/SQSTM1 protein, which normally degrade during autophagy, can be detected in the muscles of IBM and IMNM patients, suggesting dysregulation of the autophagic process of this protein (153, 154). It is known that dysregulation or excessive autophagy could lead to cell death and has been associated with several human diseases, including IBM (155). Although autophagy has not been extensively studied in other IIM phenotypes, Cappallettic et al. (156) found that, in addition to IBM, autophagy processes are also activated in PM, DM, and JDM. Girolamo et al. (157) reported a higher proportion of myofibers correlated with the presence of autophagy markers such as microtubule-associated protein light chain 3b (LC3b) and p62/SQSTM1 in muscle biopsies from patients with IMNM compared to those from patients with DM and PM.

Recent study was reported that a selected group of autophagy-related genes, such as *CCL2*, cyclin-dependent kinase inhibitor 1A (*CDKN1A*)*, FOS*, myelocytomatosis (*MYC*), and TNF superfamily member 10 (*TNFSF10*)—whose functions are related to IFN-I signaling pathways—significantly influenced the infiltration of multiple immune cells, including B-cells, macrophages, and NK cells, in samples from DM patients compared to controls, suggesting that these genes may be potential diagnostic biomarkers for DM (158).

## 7 Idiopathic inflammatory myopathies and myositis-specific antibodies: A role for endoplasmic reticulum stress?

In addition to the detection of MHC-I by immunostaining, another diagnostic tool in IIM is the presence of MSAs. Although a direct association between MSAs production and ER stress has not been defined, some reports have touched on the subject. The SRP auto-antigen might be an example of antigen released from tissue damage (159), a target of anti-SRP antibodies (160). It has been reported that auto-antibodies against SRP54 exist in IMNM and PM patients with dilated cardiomyopathy, disease severity, and remarkably high levels of muscle enzymes (160–162). One study of DM patients documented the presence of anti-SRP72 antibodies (163). Recently, a possible association between anti-calreticulin (anti-CRT) antibodies and malignancy in IIM was reported (164).

In patients seropositive for anti-cN1A, an auto-antibody commonly associated with IBM, colocalization of the cN1A auto-antigen with p62/SQSTM1 (an autophagy-related protein considered a pathological hallmark of IBM) in perinuclear regions of myofibers was found (165). Furthermore, *in vitro* and *in vivo* passive immunization models with immunoglobulin G extracted from anti-cN1A–seropositive IBM patient serum samples have been found to exhibit higher p62/SQSTM1 expression and abundant aggregations in the cytoplasm of supplemented rhabdomyosarcoma cells. Likewise, anti-cN1A–positive IBM immunoglobulin G–injected mice showed p62/SQSTM1 aggregates in myofibers (165). Concerning the involvement of ER stress as a potent inducer of autophagy, it has been hypothesized that other MSAs, such as anti-SRP, in IMNM could alter the function of SRP in the proper elongation of polypeptide chains, the induction of ER stress, and chaperone-assisted selective autophagy of defective polypeptides (154). Interestingly, both anti-SRP and anti-HMGCR auto-antibodies might have shown a pathogenic role *in vitro* because they are involved in muscle fiber atrophy associated with the increase of IL-6, TNF, and ROS as well as impaired muscle regeneration by a defect of myoblast fusion due to decreased levels of IL-4 and IL-13 (166).

Inflammatory mediators such as cytokines have been reported in the muscles of IBM patients, with a correlation between the messenger RNA expression of IL-1β and amyloid precursor protein, a protein frequently observed in rimmed vacuoles associated with the IBM phenotype. Upregulation of amyloid precursor protein and β-amyloid expression in skeletal muscle after IL-1β and IFN-γ stimulation, as well as co-localization of IL-1β and β-amyloid, has also been reported (167). Additionally, IFN-γ induces ubiquitylated inclusions in a manner independent of cell type in mouse and human cells (168). Considering that viral infections, especially by human immunodeficiency virus or human T lymphotropic virus 1, are related to IBM immunopathogenesis (169), we could infer that they facilitate an autoimmune process because of the secretion of cytokines such as IFN-γ, which promotes, in turn, the MHC-I upregulation and ubiquitylated inclusions that finally trigger ER stress responses (**Figure 1D**).

It is interesting that protein aggregates have also been reported in other autoimmune diseases, e.g., Sjögren’s syndrome, which has also been associated with the presence of the auto-antibody anti-cN1A in up to 36% of patients (27, 170). In addition to all these mechanisms, the presence of auto-antibodies can also probably cause ER stress because in patients with lupus nephritis, the anti–double-stranded DNA antibodies (anti-dsDNA) bound to a human mesangial cell induce ER stress and activation of NF-κB *via* PERK/eIF2α/ATF4 (171). Finally, it is important to recall that MSAs have a pathogenic role or are an epiphenomenon in IIM (172).

## 8 Conclusions and perspectives

Taking into account the importance of the ER for the maintenance of homeostasis of the musculoskeletal system in the regulation of proteins, there is probably a relationship between immunological, non-immunological, and infectious pathophysiological processes for the activation of ER stress and autoimmunity. An example of this might be IIMs, and, although this process is not fully understood, there are indications of the participation of cells of the immune system, auto-antibodies, viral processes, involvement of MHC-I, and cytokine signaling pathways in the activation of ER stress. However, ER stress responses have also been observed in other autoimmune diseases, as discussed above. Another special issue, which was not mentioned in this review and is important to consider in future studies, is the design of possible targeted therapies to attenuate, modulate, or eliminate ER stress in addition to the classical therapies used in IIM. Finally, it is possible to suggest that ER stress is related to the origin of autoimmune diseases and their possible consequences on auto-antibody production in IIM.

## Author contributions

Conceived and designed the idea: EC-S, EM-G, AL-B and MV-M. Conducted the bibliographic search: EC-S, EM-G, AL-B, OP-M, IG-D, EC-A, AA-V, BM-M, KA-A, JA-B, FP-V, GT-G and MV-M. Analysis and discussion of the information: EC-S, EM-G, AL-B, OP-M, IG-D, EC-A, AA-V, BM-M, KA-A, JA-B, FP-V, IG-T, AL-G, BP-Z, GT-G and MV-M. Wrote the paper: EC-S, EM-G, AL-B, OP-M, EC-A, AA-V, and MV-M. Figure editing: EM-G, and AL-B. All authors contributed to the article and approved the submitted version.

## Conflict of interest

The authors declare that the research was conducted in the absence of any commercial or financial relationships that could be construed as a potential conflict of interest.

## Publisher’s note

All claims expressed in this article are solely those of the authors and do not necessarily represent those of their affiliated organizations, or those of the publisher, the editors and the reviewers. Any product that may be evaluated in this article, or claim that may be made by its manufacturer, is not guaranteed or endorsed by the publisher.
